# Trends in antimicrobial resistance in Israel, 2014–2017

**DOI:** 10.1186/s13756-019-0535-1

**Published:** 2019-06-04

**Authors:** Yaakov Dickstein, Elizabeth Temkin, Michal Ish Shalom, David Schwartz, Yehuda Carmeli, Mitchell J. Schwaber

**Affiliations:** 1 National Center for Infection Control, Ministry of Health, Weizmann 6, 6423906 Tel Aviv, Israel; 2 Sackler Faculty of Medicine, Tel Aviv University, Kalechkin 35, 6997801 Tel Aviv, Israel

**Keywords:** Antibiogram, Antibiotic, Bloodstream isolate, CRE, ESBL, Israel, MRSA, Resistance, Surveillance, VRE

## Abstract

We analyzed Israeli national data on antimicrobial susceptibility from bloodstream isolates collected between 2014 and 2017 and compared resistance proportions with those of Europe. The incidence of bloodstream infection (BSI) caused by most antibiotic-resistant organisms remained unchanged or decreased. An exception was increased incidence of BSI caused by third-generation cephalosporin-resistant *Escherichia coli*. Overall, resistance proportions were similar to those observed in southern Europe, with the exception of a lower proportion of carbapenem-resistant *Klebsiella pneumoniae* in Israel.

The Israel National Center for Infection Control (NCIC), a unit of the Ministry of Health (MoH), has been monitoring antimicrobial resistance in selected bacteria in hospital bloodstream isolates since 2013 in the context of a national infection control program [1]. As part of the program, all acute-care hospitals in Israel are required to submit a monthly report to the NCIC with isolate-level data on all blood cultures growing seven sentinel bacteria. These data are utilized to provide monthly feedback to the hospitals on the incidence of bacteremia with resistant organisms and annually to generate institutional- and national-level antibiograms. The present report presents surveillance data on antimicrobial susceptibility testing in Israel in the period 2014–2017 and compares them with similar data from Europe.

## Antimicrobial resistance surveillance and reporting

Data on antimicrobial resistance in bloodstream bacterial isolates were collected from inpatient medical records in all departments in Israel’s acute-care hospitals. An isolate was considered resistant when reported by the hospital’s clinical microbiology laboratory as intermediately susceptible (I) or resistant (R). For antibiotic categories combining more than one antibiotic agent, resistance to any agent within a class was regarded as resistance to the class.

Data on blood isolates were collated and an antibiogram prepared for selected bacteria of epidemiological importance. All hospitals contributed data on all organisms for each year. For each organism, only the first isolate per patient per year was considered. Data on colistin resistance for 2017 were not reported in accordance with the European Committee on Antimicrobial Susceptibility Testing (EUCAST) warning concerning results of susceptibility testing [2]. Data on population size were taken from the World Bank [3] while annual patient days were taken from statistics regularly distributed by the MoH [4–7]. Data used for comparison were taken from the 2017 report published by the European Antimicrobial Resistance Surveillance Network (EARS-Net) [8]. The Cochran-Armitage test was used to test for significant changes over time in the proportion of isolates that were resistant. We tested for significant changes over time in bloodstream infection (BSI) incidence using Poisson regression or negative binomial regression (when data were overdispersed). All calculations were performed with STATA v14.2 (StataCorp LLC, College Station, TX).

## Surveillance findings

Data on the incidence of bloodstream infection (BSI) and the proportion of isolates that were resistant are presented in Tables 1 and 2. The following trends were observed:

- A decrease in the incidence of all *Acinetobacter baumannii* BSI (14.5 to 12.1 per 100,000 patient-days, *p* < 0.001) and the incidence of carbapenem-resistant *A. baumannii* BSI (11.5 to 9.5 per 100,000 patient-days, *p* = 0.001), while the proportion of *A. baumannii* isolates resistant to carbapenems remained stable at 75–80%.
- An increase in the incidence of all *Enterococcus faecium* BSI (6.5 to 8.2 per 100,000 patient-days, *p* = 0.001), while the incidence of vancomycin-resistant *Enterococcus faecium* (VREf) BSI remained unchanged (1.7 to 1.6 per 100,000 patient-days, *p* = 0.60). The proportion of *E. faecium* isolates resistant to vancomycin decreased (25.6 to 19.0%, *p* = 0.020).
- Increases in the incidence of all *Escherichia coli* BSI (101.6 to 110.9 per 100,000 patient-days, *p* < 0.001), 3rd-generation cephalosporin (3GC)-resistant *E. coli* BSI (24.1 to 32.4 per 100,000 patient-days, *p* < 0.001), and the proportion of *E. coli* isolates resistant to 3GC (29.5 to 32.0%, *p* = 0.017). The proportion of *E. coli* isolates with combined resistance to 3GC, fluoroquinolones and aminoglycosides decreased from 10.6 to 8.3% (*p* < 0.001); the incidence of BSI caused by these pathogens did not change significantly (8.7 to 8.2 per 100,000 patient-days (*p* = 0.40).
- No significant change in the incidence of all *Klebsiella pneumoniae* BSI (46.3 to 47.4 per 100,000 patient-days, *p* = 0.35), carbapenem-resistant *K. pneumoniae* BSI (2.1 to 1.9 per 100,000 patient-days, *p* = 0.58) or in the proportion of carbapenem-resistant isolates (4.6 to 4.0%, *p* = 0.36). The proportion of *K. pneumoniae* isolates with combined resistance to 3GC, fluoroquinolones and aminoglycosides decreased from 23.7 to 19.2% (*p* = 0.002) while the incidence of BSI caused by these strains remained unchanged (8.0 per 100,000 patient-days, *p* = 0.80).
- The incidence of *Pseudomonas aeruginosa* BSI increased (22.2 to 25.7 per 100,000 patient-days, *p* < 0.001), while the incidence of carbapenem-resistant *P. aeruginosa* remained unchanged (3.5 to 3.3 per 100,000 patient-days, *p* = 0.72). The observed decrease in the proportion of carbapenem-resistant isolates did not achieve statistical significance (16.0 to 13.0%, *p* = 0.057).
- The incidence of all *Staphylococcus aureus* BSI increased, from 42.4 to 47.1 per 100,000 patient-days (*p* < 0.001), driven by the rising incidence of methicillin-susceptible *S. aureus* BSI (26.2 to 32.7 per 100,000 patient-days, *p* < 0.001). The incidence of BSI caused by methicillin-resistant *S. aureus* (MRSA) declined from 16.2 to 14.4 per 100,000 patient-days (*p* = 0.04). The percentage of methicillin-resistant *S.aureus* isolates decreased from 38.2 to 30.6% (*p* < 0.001).
- The combined incidence of *A. baumannii*, *E. faecium*, *K. pneumoniae* and *P. aeruginosa* BSI remained unchanged (89.5 to 93.4 per 100,000 patient-days, *p* = 0.14), as did the combined incidence of VREf and carbapenem-resistant *A. baumannii*, *K. pneumoniae* and *P. aeruginosa* (18.8 to 16.3 per 100,000 patient-days, *p* = 0.090).

**Table 1:**
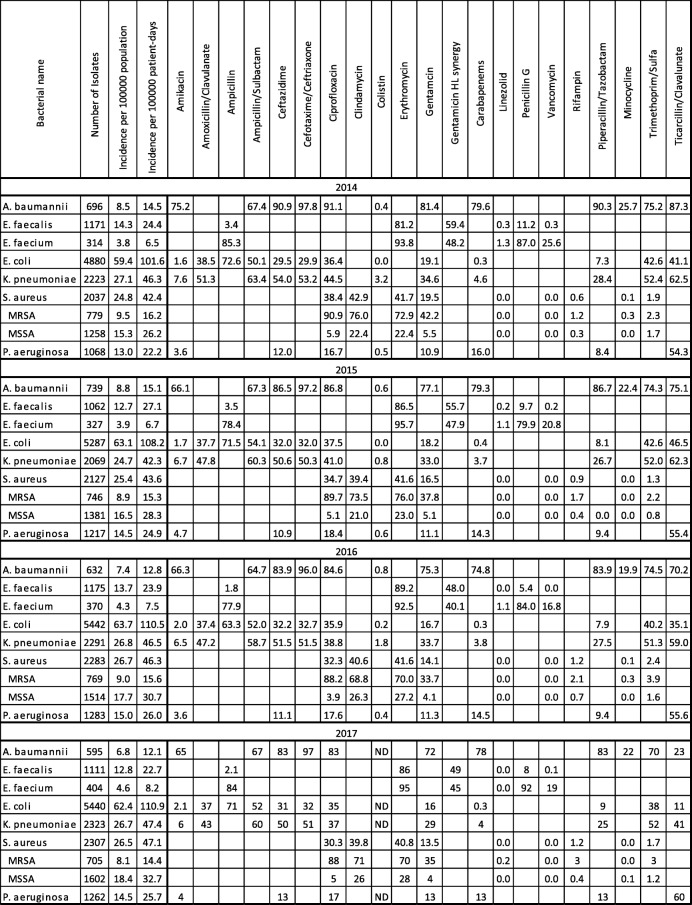
Percentage of Resistant Isolates, Israel, 2014–2017

*HL* High level, *MRSA* methicillin-resistant *S. aureus*, *MSSA* methicillin-susceptible *S. aureus*, *ND* No data, *Sulfa* Sulfamethoxazole

**Table 2:** Percentage and Incidence of Combined Resistance Phenotypes, Israel, 2014–2017

	2014		2015		2016		2017	
Bacterial name	Percentage of resistant isolates	Incidence per 100,000 patient-days	Percentage of resistant isolates	Incidence per 100,000 patient-days	Percentage of resistant isolates	Incidence per 100,000 patient-days	Percentage of resistant isolates	Incidence per 100,000 patient-days
A. baumannii Carb+FQ + AG	73.2	8.4	74.2	9.3	72.2	7.8	70.1	7.2
*E. coli* 3GC	29.5	24.1	32.0	30.2	32.4	32.3	32.0	32.4
E. coli 3GC + FQ + AG	10.6	8.7	10.3	9.7	9.3	9.1	8.3	8.2
*K. pneumoniae* 3GC + FQ + AG	23.7	8.0	20.8	7.6	20.7	8.3	19.2	8.0
K. pneumoniae Carb+Col	1.8	0.2	0.6	0.0	0.4	0.0	ND	ND

*AG* Aminoglycoside-resistant, *Carb* Carbapenem-resistant, *Col* Colistin-resistant, *FQ* Fluoroquinolone-resistant, *3GC* 3rd-generation cephalosporin-resistant

In general, resistance proportions were similar to those reported by countries in southern Europe (Italy and Greece), with the exception of carbapenem-resistant and combined 3GC-, fluoroquinolone- and aminoglycoside-resistant *K. pneumoniae*, for which the proportions were lower in Israel (4.0%/19.2%, respectively, in 2017, vs 29.7/31.6%% in Italy and 64.7/47.9%% in Greece) and carbapenem-resistant *P. aeruginosa*, for which the proportion was lower in Israel (13.0%) than in Greece (39.3%) [8].

## Discussion

In Israel between 2014 and 2017, the incidence of BSI caused by most antibiotic-resistant organisms remained unchanged or decreased. An exception was the incidence of BSI caused by 3GC-resistant *E. coli*, which increased. Overall, resistance proportions were similar to those observed in southern Europe, with the exception of carbapenem-resistant *K. pneumoniae*, for which the resistance proportion in Israel was markedly lower.

Following an outbreak of carbapenem-resistant Enterobacteriaceae (CRE) in Israeli health-care facilities in 2006, a national intervention was implemented to identify carriers of CRE and prevent ongoing transmission [9]. The intervention succeeded in reducing the incidence of CRE acquisition and subsequently overall CRE prevalence in Israel [10–12]. This success catalyzed a broader nationwide infection control (IC) program which, apart from surveillance of antimicrobial susceptibility, included the establishment of a central reference laboratory, enhancement of IC programs within individual healthcare institutions and the creation of a national antibiotic stewardship program [1]. Thus, the trends toward decreasing incidence of many antibiotic-resistant organisms reported here occurred in the context of an intensive, nationally-coordinated IC effort which included the use of antimicrobial susceptibility data to plan and prioritize focused interventions where warranted.

This report has a number of limitations. First, there is no national standard for reporting of antimicrobial susceptibility. Although most microbiology laboratories in Israel employ Clinical and Laboratory Standards Institute (CLSI) breakpoints, this is not mandatory. Thus, it is possible that for certain antibiotic categories for which CLSI breakpoints are higher than those issued by the European Committee on Antimicrobial Susceptibility Testing (EUCAST), levels of antimicrobial resistance as we reported are lower than they would be utilizing EUCAST criteria. However, given that differences where present tend to be relatively small (i.e. one dilution), we believe that the general comparison with EARS-Net data remains valid [13]. Second, we do not differentiate resistance levels between community-acquired and hospital-acquired/health care-associated infections. Thus, is it likely that for nosocomial infections, the resistance levels are higher than what we have reported.

As noted above, the success of the intervention for CRE, which saw the proportion of carbapenem-resistant *K. pneumoniae* bloodstream isolates decline from 22% in 2007 to 4% one decade later [1], can explain the difference in the proportion of carbapenem-resistant *K. pneumoniae* BSI isolates between Israel and southern Europe. In contrast, both our data and previous Israeli studies indicate a steady increase in the incidence of bloodstream infections due to extended-spectrum β-lactamase (ESBL)-producing Enterobacteriaceae and the proportion of Enterobacteriaceae blood isolates producing ESBL during the same period [14–16]. The use of targeted control efforts for CRE has been cited as one factor explaining the limited spread of CRE in the United States as compared with ESBL-producing bacteria [17].

An important difference in the epidemiology of ESBL-producing and carbapenem-resistant Gram-negative bacteria is the role of asymptomatic carriers in the community. Carbapenem-resistance is primarily nosocomial, with little to no transmission in community settings reported outside of eastern Asia [18]. ESBL-producing Enterobacteriaceae, however, have spread to colonize healthy community dwellers with no or minimal healthcare contact [19]. These carriers serve as a major reservoir for infections with ESBL-producing bacteria in hospitals as well, implying that interventions which concentrate principally on healthcare settings may have limited success in controlling the spread of these organisms [20, 21]. In Israel, where interventions have focused on healthcare facilities, the combined incidence of BSI with typically hospital-acquired antibiotic-resistant organisms decreased at the same time that incidence of ESBL-producing *E. coli* BSI rose. Ongoing efforts are necessary to further inhibit the spread of resistant organisms, including the development of interventions aimed at community settings.

## Abbreviations

3GC: 3rd-generation cephalosporin
BSI: Bloodstream infection
CLSI: Clinical and Laboratory Standards Institute
CRE: Carbapenem-resistant Enterobacteriaceae
EARS-Net: European Antimicrobial Resistance Surveillance Network
ESBL: Extended-spectrum β-lactamase
EUCAST: European Committee on Antimicrobial Susceptibility Testing
I: Intermediate susceptibility
IC: Infection control
MoH: Ministry of Health
MRSA: Methicillin-resistant *Staphylococcus aureus*
NCIC: National Center for Infection Control
R: Resistant
VREf: Vancomycin-resistant *Enterococcus faecium*
